# Multistage Classification of Current Density Distribution Maps of Various Heart States Based on Correlation Analysis and k-NN Algorithm

**DOI:** 10.3389/fmedt.2021.779800

**Published:** 2021-12-08

**Authors:** Yevhenii Udovychenko, Anton Popov, Illya Chaikovsky

**Affiliations:** ^1^Electronic Engineering Department, Faculty of Electronics, Kyiv Polytechnic Institute, Kyiv, Ukraine; ^2^Institute of Cybernetics, National Academy of Sciences of Ukraine, Kyiv, Ukraine

**Keywords:** magnetocardiography, current density imaging, current density distribution map, k-NN classification, ischemic heart disease, heart failure diagnostics, correlation analysis

## Abstract

Magnetocardiography is a modern method of registration of the magnetic component of electromagnetic field, generated by heart activity. Magnetocardiography results are a useful source for the diagnosis of various heart diseases and states, but their usage is still undervalued in the cardiology community. In this study, a two-stage classification by correlation analysis using a k-Nearest Neighbor (k-NN) algorithm is applied for the binary classification of myocardium current density distribution maps (CDDMs). Fourteen groups of CDDMs from patients with different heart states, healthy volunteers, sportsmen, patients with negative T-peak, patients with myocardial damage, male and female patients with microvascular disease, patients with ischemic heart disease, and patients with left ventricular hypertrophy, divided into five and three different groups depending on the degree of pathology, were compared. Selection of best metric, used in classifier and number of neighbors, was performed to define the classifier with best performance for each pair of heart states. Accuracy, specificity, sensitivity, and precision values dependent on the number of neighbors are obtained for each class. The proposed method allows to obtain a value of average accuracy equal to 96%, 70% sensitivity, 98% specificity, and 70% precision.

## Introduction

Magnetocardiography (MCG) is a non-invasive and risk-free technique of measuring magnetic field generated by the electrical activity of the heart using extremely sensitive devices, such as a superconducting quantum interference device. MCG can be applied along with electrocardiography (ECG) to get the information on the state of a patent's cardiovascular system and provides additional opportunities for visualization and analysis of obtained data (1), although at the moment most efforts in MCG signal processing are made mostly for its informative representation (2, 3).

Magnetocardiographic mapping is performed for diagnostics of ischemic heart disease (4), Wolf-Parkinson's-White syndrome (5), and other heart failures associated with current flow changes in heart muscles. Fairly good results have been achieved in the classification of current density vector maps based on clusters analysis for coronary artery disease and ischemic heart disease detection in patients with normal or unspecifically changed ECG (6) and echocardiogram (7). Nevertheless, the classification of MCG data, especially current density distribution maps (CDDMs), is on its starting point and is applied only for specific disease diagnostics.

In our previous study, we applied different technics, such as decision trees, support vector machine, and k-NN with different metrics for multiclass classification and obtained accuracy in the range of 55–65% (8), which is not satisfactory for wider clinical applications. In our further study, we developed a multiclass classifier based on correlation analysis (9), with value of accuracy equal to 95%, in order to provide a tool for the complex analysis of a patient's cardiovascular system by MCG. In other study, we applied a k-NN algorithm for the binary classification of CDDMs for ischemic heart disease recognition (10). Developed classifiers showed accuracy in the range of 60–90% depending on the type of CDDM. In other study, we developed a k-NN classifier in order to distinguish normal heart state from heart failures, such as negative T-peak and microvessels (diffuse) abnormalities (11). Additionally, we advanced our algorithms to reach higher classification performance.

The aim of this study is to develop a multistage classifier by combining two methods: correlation analysis as the 1st stage, performed for multiclass classification, and k-NN classification as the 2nd stage to make the result of previous stage more accurate.

## Data Collection and Method Description

### Formation of CDDMs Using MCG Data

For spatial data capture during MCG measurements, observation points are the intersection nodes of a square grid, which is binding to anatomical landmarks on the thorax. As the number of nodes is limited, in order to localize areas of pathological activity of the myocardium and to build maps of instant distribution of magnetic field induction in the heart, smooth filling and interpolation of the function of two variables in the points beyond the bounds of a standard grid are usually performed. Thus, instant magnetic field distribution maps are built using two-dimensional interpolation algorithms and based on synchronous averaged MCG curves. Then, the MCG inverse problem is solved, and the map of magnetic field distribution can be converted into an instant CDDM (12).

**Figure 1:**
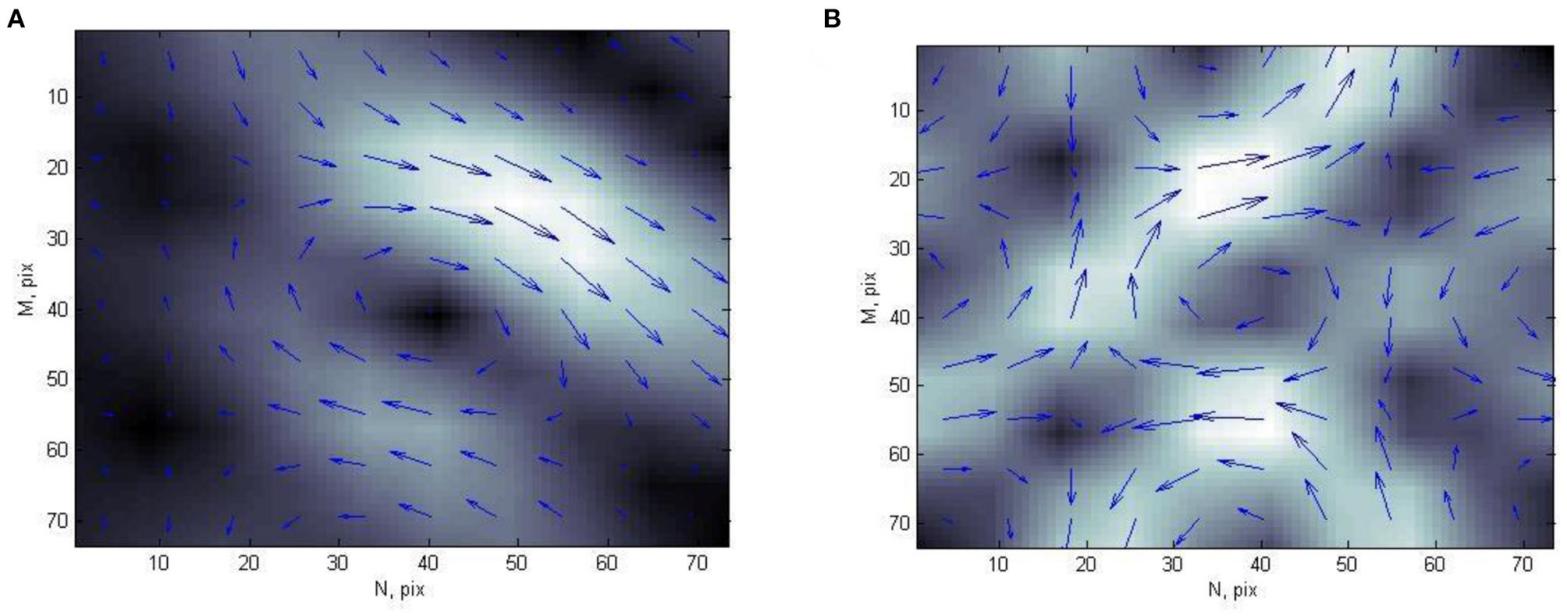
Example of current density distribution map of **(A)** person with normal heart state and **(B)** patient with ischemic heart disease.

Each CDDM in this study is formed by transformation of current density vectors, obtained from MCG. Each CDDM is a grayscale discrete image with dimensions of M × N pixels, where white color corresponds to the highest brightness, and the black color corresponds to the lowest brightness (Figure 1). Thus, the brightness of an image corresponds to the current density value in a particular point.

In most cases, CDDMs are calculated for precise time instants with some steps (up to 10 ms) during T wave of electrocardiogram QT interval. This enables a researcher to associate ECG data with corresponding characteristics calculated from CDDMs. Each CDDM is normalized by maximal value (13).

### Multiclass Classification Based on Correlation Analysis

The main idea of the method of current density distribution map classification based on correlation analysis is to find and compare the correlation coefficients of the map under analysis with each of the maps in the reference set. Reference set consist of pre-classified by the doctor current density distribution maps, each of which belongs to one of the groups corresponding to a certain state of the cardiovascular system. For each of the classified maps, the correlation coefficients of the vector of values and the vector of directions with the corresponding vectors of each of the maps from the reference set are calculated as follows:


(1) $$\begin{array}{l} r=\;\frac{\sum_{i=1}^{n}\left(x_{i}-\bar{x}\right)\left(y_{i}-\bar{y}\right)}{\sqrt{\sum_{i=1}^{n}{\left(x_{i}-\bar{x}\right)}^{2}}\sqrt{\sum_{i=1}^{n}{\left(y_{i}-\bar{y}\right)}^{2}}} \end{array}$$

Where *n* is the dimension of the vectors, for our case *n* = 100,

*x*_*i*_, and *y*_*i*_ are values of vectors for which the correlation coefficient is calculated and

$\bar{x}$, and $\bar{y}$ are the mean values of the vectors, calculated as follows:


(2) $$\begin{array}{l} \bar{x}=\;\frac{1}{n}\overset{n}{\underset{i\;=\;1}{\sum }}x_{i} \end{array}$$

(3) $$\begin{array}{l} \bar{y}=\;\frac{1}{n}\overset{n}{\underset{i\;=\;1}{\sum }}y_{i} \end{array}$$

After that, the values of the obtained correlation coefficients for two vectors are multiplied; thus, the resulting correlation coefficient is obtained, which takes into account both the modulus correlation and the direction of the current density vectors. As a result, a set of the resulting correlation coefficients with the maps of each group of the reference set is obtained for each map. After that, an array of *m* maximum values of the resulting correlation coefficient is formed for each group, and their average value is found. Thus, for each CDDM we obtain a set of key-value pairs with the groups corresponding to the state of the cardiovascular system as keys, and the abovedescribed average values of the maximum correlation coefficients as corresponding values. The maximum of these values indicates the group to which the map of the distribution of the current density to be classified should be assigned (9). The best result was obtained for *m* in the range of 1–5, and the accuracy of classification in these cases is highest and does not significantly depend on the number of maximum values; therefore, in this study, we use *m* = 3.

### k-Nearest Neighbor Algorithm

One of the methods for pattern classification is the k-nearest neighbor (k-NN) rule. It classifies each unlabeled object according to the majority label of its k-nearest neighbors in the training set. Despite its simplicity, the k-NN rule often yields competitive results and in certain domains, when cleverly combined with prior knowledge, it can help to solve even quite difficult classification tasks.

The result of k-NN classification depends significantly on the metric used to compute distances among different feature vectors. In (12), it was shown that using different distances for k-NN classification gives opportunity to decrease the error rates for different classification problems, such as face recognition, spoken letter recognition, and text categorization. It was also demonstrated that a k-NN classifier with correctly chosen distance metric shows better result, even when compared to SVM used for same classification tasks.

In this study, three most commonly used metrics, which are special cases if Minkowski distance, Eucledian, Cityblock, and Chebychev, were examined. Let us consider X as a 1-×-32 feature vector of a classified CDDM and Y as a feature vector of each CDDM in the training set. In our study, binary classifiers with three different distance metrics were developed. A classifier with an Eucledian metric distance between two points Xs and Yt, whose coordinates are values of X and Y, respectively, is defined as follows:


(4) $$\begin{array}{l} d_{st}^{2}=\left(x_{s}-y_{t}\right){\left(x_{s}-y_{t}\right)}^{{\;}^{\prime }} \end{array}$$

For Cityblock (also known as Manhattan) metric:


(5) $$\begin{array}{l} d_{st}=\overset{n}{\underset{j\;=\;1}{\sum }}|x_{sj}-y_{tj}| \end{array}$$

where n is the size of vectors X and Y, and in our case *n* = 32–number of features.

For Chebychev metric:


(6) $$\begin{array}{l} d_{st}=\{|x_{sj}-y_{tj}|\} \end{array}$$

## Experimental Results

### Experiment Setup and Data Preparation

For this study, 2,142 CDDMs from 14 groups of patients with different heart states were used. These groups were the following: healthy volunteers (“NORM1” class) represented by 24 patients, sportsmen (“NORM2” class)−36 patients, patients with myocardial damage–six patients, patients with non-coronagenic diseases−27 persons, male (21 patients) and female (18 patients) patients with microvascular disease, patients with ischemic heart disease, and patients with left ventricular hypertrophy, divided into five and three different groups depending on degree of pathology, where group “CAD 1” represents the lightest and “CAD 5” the hardest form of Ischemic heart disease, “LVH 1” the lightest, and “LVH 3” the hardest form of left ventricular hypertrophy. To group CAD 1 belong 21 persons, CAD 2−19, CAD 3−18, CAD 4−9, CAD 5−13, LVH 1−10, LVH 2−14, LVH 3−11. Each patient in these groups is characterized by CDDMs of specific structure. The structure of CDDM in our understanding is the mutual location of zones with high and low density of currents.

### Rules for Proceeding to the 2nd Stage of Classification

**Figure 2:**
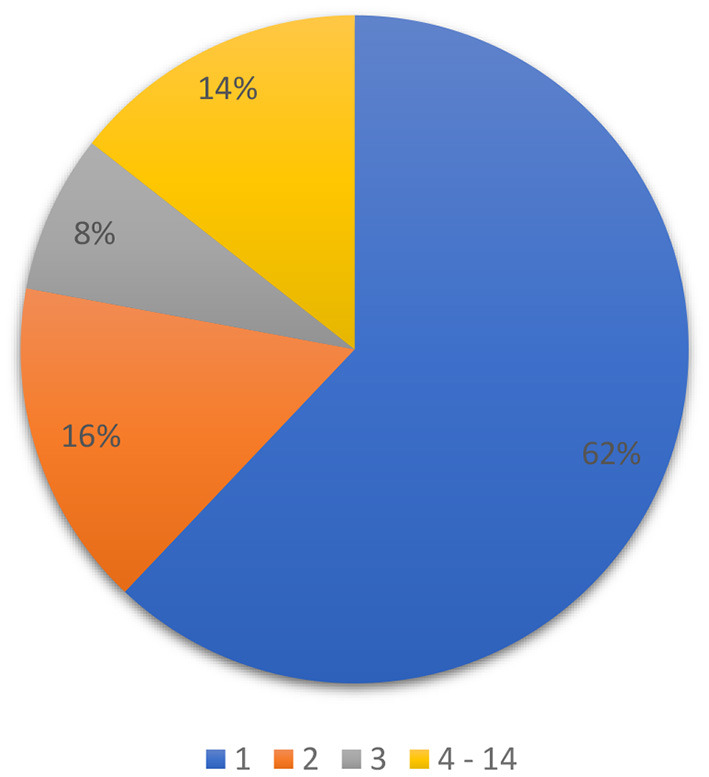
Distribution of the right group among predicted groups in set, ordered by correlation coefficient in descending order.

In order to design the 2nd stage of multistage classifier, the result of multiclass classification, which is the 1st stage of our multistage classifier, was analyzed. A map of resulting correlation coefficients and corresponding groups for each CDDM was ordered in a descending manner: the 1st group with the highest correlation value and 14th with the lowest. After that, we compared all these groups with actual group, to which CDDM belongs, to define the place of right group in the ordered set. The result of this analysis is shown in Figure 2. As we can see, 86% of the right groups can be found among the first three groups with highest correlation coefficients. This information is significant for us, as it allows us to concentrate on the three groups with highest correlation in the 2nd stage of classification.

**Figure 3:**
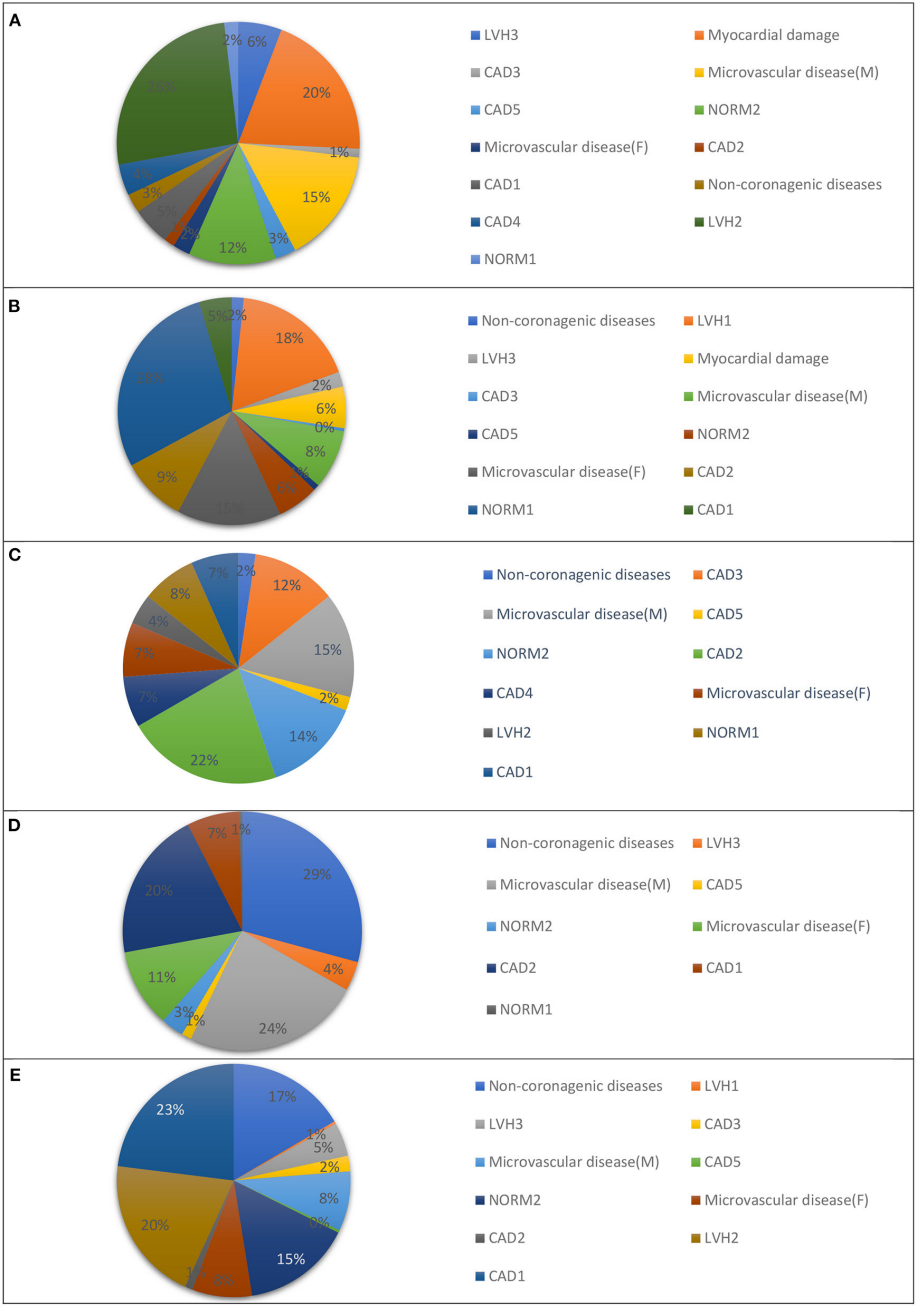
Groups that are most often misclassified to: **(A)** LVH1, **(B)** LVH2, **(C)** LVH3, **(D)** IHD3, and **(E)** NORM1 using a multiclass classifier based on correlation analysis.

**Figure 4:**
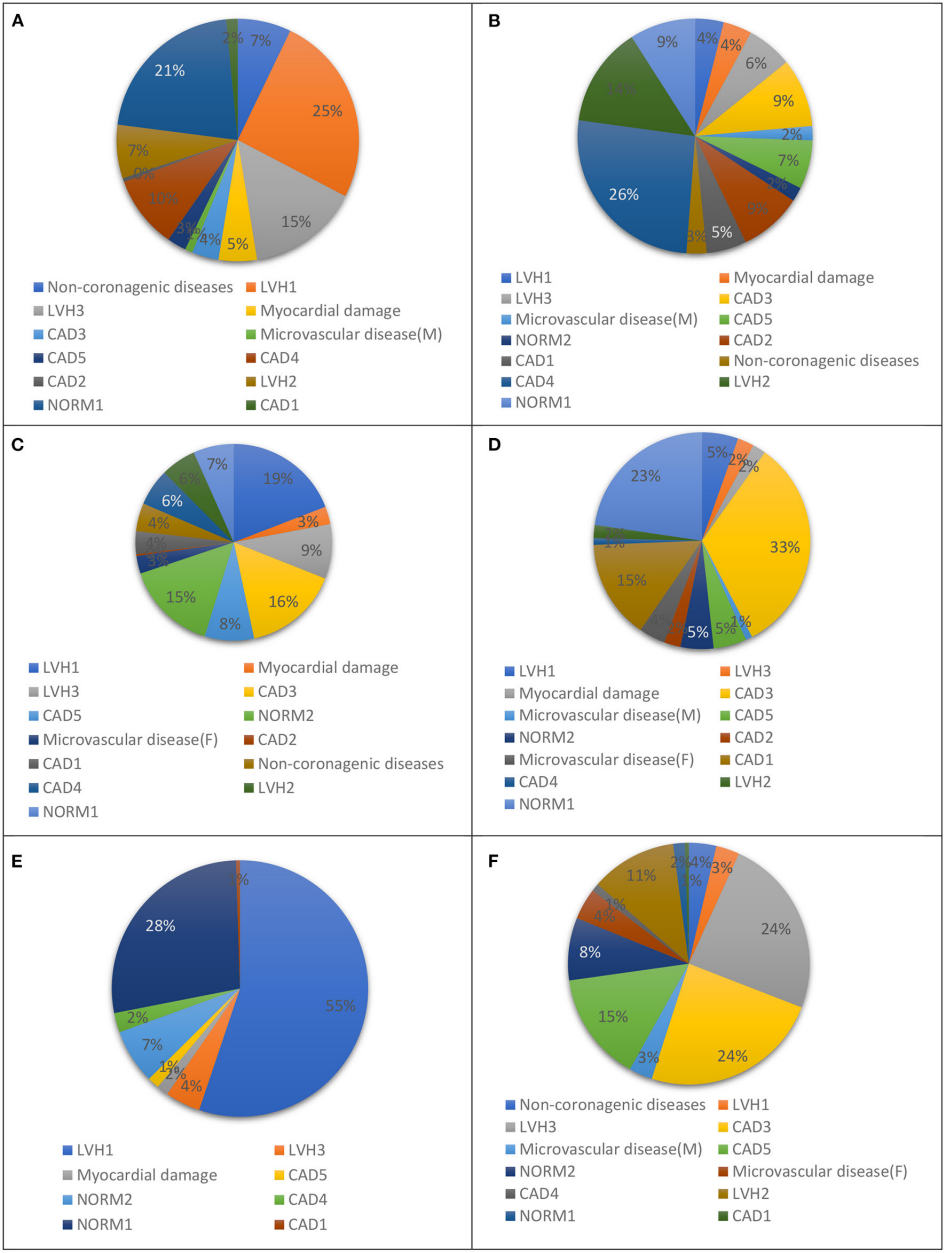
Groups to which: **(A)** NORM2, **(B)** DIFFf, **(C)** DIFFm, **(D)** REUMO, **(E)** LVH2, and **(F)** IHD2 are misclassified most often using a multiclass classifier based on correlation analysis.

In our previous study, we presented a multiclass classifier that has, in general, good characteristics (9); however, classification showed poor precision for groups LVH1, LVH2, LVH3, CAD3, and NORMAL. It means a lot false positive results for these groups. At the same time, the classifier shows low sensitivity for the groups Microvascular disease (M), Microvascular disease (F), NORM2, Non-coronarogenic diseases, LVH2, CAD2, which means a lot of false negative results for these groups. Misclassifications of CDDMs from the above-mentioned groups with low precision or sensitivity dramatically affect the characteristics of the classifier in general, so the main goal of the 2nd stage of classification is to reduce number of false positive results for groups with low precision and the number of false negative results for groups with low sensitivity. To do that we analyzed the groups to which belongs CDDMs that are misclassified to groups with low precision and groups to which are misclassified CDDMs from groups with low sensitivity. The result of the analysis is shown in Figures 3, 4.

According to obtained results, the list of groups that are misclassified to groups with low precision most often was formed. These groups are presented in Table 1.

**Table 1:** Groups with low precision and corresponding groups that are misclassified to it most often.

LVH1	Myocardial damage, LVH2, Microvascular disease (M), NORM2
LVH2	NORM1, LVH1, Microvascular disease (F)
LVH3	CAD2, Microvascular disease (M), CAD3, NORM2
CAD3	Non-coronarogenic diseases, CAD2, Microvascular disease (M), Microvascular disease (F)
NORM1	CAD1, LVH2, NORM2, Non-coronarogenic diseases

List of groups to which groups with low sensitivity are misclassified most often are presented in Table 2.

**Table 2:** Groups with low sensitivity and corresponding groups to which they are misclassified most often.

NORM2	LVH1, LVH3, NORM1
Microvascular disease (F)	CAD4, LVH2
Microvascular disease (M)	CAD1, CAD3, NORM2
Non-coronarogenic diseases	CAD1, CAD3, NORM1
LVH2	LVH1, NORM1
CAD2	LVH2, LVH3, CAD3, CAD5

The obtained result allows us to describe the rules for our two-stage classifier: 2nd stage of classification (k-NN classification) is needed if one of the conditions is met:

1) Group with the highest correlation belongs to one of the groups with low precision (LVH1, LVH2, LVH3, CAD3, or NORM1) and the 2nd or 3rd group belongs to one of the corresponding groups from in Table 1.
2) Group with the highest correlation belongs to one of the groups with low sensitivity [NORM2, Microvascular disease (F), Microvascular disease (M), NON-CORONAROGENIC DISEASES, LVH2, or CAD2] and the 2nd or 3rd group belongs to one of the corresponding groups in Table 2.

### Feature Extraction and Parameter Definition for the 2nd Stage

**Figure 5:**
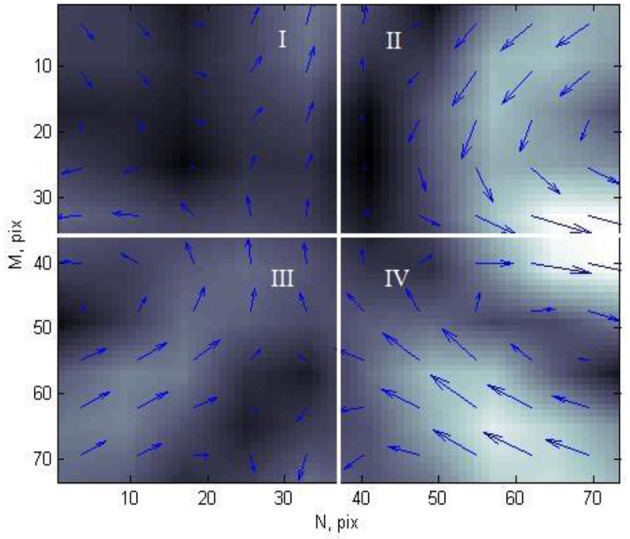
Current density distribution map of patient with ischemic heart disease, divided into four parts.

In this study, for the 2nd stage, each CDDM is divided into four equal parts (quarters), as shown in Figure 5.

Each element of CDDM has two parameters: brightness, which corresponds to the current density in a corresponding point, and angle of magnetic field vector in each point. For these two sets of data, in each quarter, the following characteristics were calculated: mean value of the elements, variance, kurtosis, and skewness of the elements. Thus, as each CDDM is divided into four parts, for which eight values are calculated (four for brightness and four for angles), each map has 32 features.

A k-NN classifier with three different metrics, described in the “k-Nearest Neighbor Algorithm” section, was applied to every pair of heart. This operation was made for different numbers of neighbors in the range of 1–15, with the purpose of finding the optimal number of neighbors to obtain best performance characteristics. For cross-validation, 20 iterations of classification were made for each heart states pair with each metric and each value of nearest neighbors. In each iteration, from the general set of CDDMs, 280 CDDMs were taken, 20 for each group. These CDDMs formed the reference set. In the same way, in each iteration, an experimental set was formed. Such approach is caused by the limitations of the general set, related with different amounts of CDDMs in groups, so we had to take into account the group with minimal number of CDDMs to form a balanced sample of maps for the experiment. Binary classification was performed for each pair of CDDMs in each pair of groups representing the heart state from the experimental set by counting distance to each CDDM from corresponding groups in the reference set. Then, the decision on the group to which CDDM should be assigned is made based on prevailing group among a defined number of neighbors.

After the experiment, we obtained the best metric and NN parameter for each pair of groups. The result is shown in Table 3. For most of the cases, the best metric is Cityblock with NN = 1.

**Table 3:** Parameters for best performance of k-Nearest Neighbor (k-NN) classification for different group pairs.

**Group 1**	**Group 2**	**Metric**	**NN**
CAD2	LVH3	Manhattan	3
Microvascular disease (M)	CAD3	Manhattan	7
Microvascular disease (F)	CAD3	Manhattan	4
CAD2	CAD3	Manhattan	5
CAD2	NORM2	Manhattan	11
Non-coronarogenic diseases	NORM2	Manhattan	5
CAD3	Non-coronarogenic diseases	Manhattan	3
CAD1	NORM2	Eucledian	5
Microvascular disease (F)	CAD2	Manhattan	10
Microvascular disease (M)	NORM1	Manhattan	12
CAD1	CAD2	Chebyshev	7
LVH2	NORM1	Manhattan	3
CAD2	Non-coronarogenic diseases	Manhattan	3
LVH1	LVH3	Manhattan	3
CAD3	NORM1	Manhattan	3
LVH3	NORM1	Manhattan	3
NORM1	NORM2	Manhattan	7
CAD2	CAD4	Manhattan	4
CAD4	CAD5	Chebyshev	5
CAD2	NORM1	Manhattan	5
LVH1	CAD3	Manhattan	3
LVH1	Myocardial damage	Manhattan	3
Myocardial damage	Non-coronarogenic diseases	Manhattan	5
OTHER PAIRS		Cityblock	1

### Experiment Setup

After analysis and definitions of rules and best parameters for each stage, 2-stage classifier can be designed. Classification of each CDDM will consist of the following steps:

1) First stage: multiclass classification by correlation analysis, as described above. As a result, a set of groups with corresponding correlation, sorted in descending order by correlation coefficient, is formed. After that, depending on the result, there are three possible options:
  - group with the highest correlation belongs (1st in result set) to one of the groups with low precision (LVH1, LVH2, LVH3, CAD3, or NORM1), and the 2nd or 3rd group belongs to one of the corresponding groups in Table 1 – proceed to 2nd stage of classification;
  - group with the highest correlation belongs to one of the groups with low sensitivity [NORM2, Microvascular disease (F), Microvascular disease (M), Non-coronarogenic diseases, LVH2, or CAD2] and the 2nd or 3rd group belongs to one of the corresponding groups in Table 2—proceed to 2nd stage of classification;
  - all other cases−2^nd^ stage is not needed. CDDM is classified to group with the highest correlation coefficient.
3) Second stage: If the 1st stage shows that additional classification is needed, k-NN classification is performed between 1st group in set and 2nd or 3rd using the corresponding parameters for this pair. After that, CDDM is classified by the result of the k-NN classification.

For cross-validation, 20 iterations of classification were made for each heart states pair with each metric and each value of nearest neighbors. In each iteration from the general set of CDDMs, 280 CDDMs were taken, 20 for each group. These CDDMs formed the reference set. In the same way, in each iteration, an experimental set was formed.

To evaluate the classification result, the following parameters were used for each group using 1-vs-all strategy: sensitivity (TPR), specificity (SPC), precision (PPV), and accuracy (ACC). These values are defined as follows:


(7) $$\begin{array}{l} TPR=\;\frac{TP}{P} \end{array}$$

(8) $$\begin{array}{l} SPC=\frac{TN}{N} \end{array}$$

(9) $$\begin{array}{l} PPV=\frac{TP}{TP+FP} \end{array}$$

(10) $$\begin{array}{l} ACC=\frac{TP+TN}{P+N} \end{array}$$

where TP is the number of CDDMs of persons with heart failure identified correctly; TN is the number of CDDMs of persons without heart failure identified correctly; P is the total amount of CDDMs of persons with heart failure; N is the total amount of CDDMs of persons without heart failure.

After all iterations, the mean value of the characteristic parameters for each classifier with a different number of neighbor value was counted.

Average accuracy was counted to evaluate the overall performance of the classifier (14):


(11) $$\begin{array}{l} ACC_{AV}=\frac{\sum_{i\;=\;1}^{l}\frac{tp_{i}+tn_{i}}{tp_{i}+tn_{i}+fp_{i}+fn_{i}}}{l} \end{array}$$

where *tp*_*i*_ is the true positive counts, *tn*_*i*_ is the true negative counts, *fp*_*i*_ is the false positive counts, *fn*_*i*_ is the false negative counts, and *l* is the number of classes (14 in our case).

### Experimental Results and Discussion

The averaged characteristic values for each group of the described experiment are presented in Table 4.

**Table 4:** Averaged two-stage classification characteristic values for each group.

**Group**	**Accuracy**	**Precision**	**Sensitivity**	**Specificity**
LVH1	0.955	0.629	0.900	0.959
LVH3	0.960	0.666	0.873	0.966
Myocardial damage	0.979	0.848	0.863	0.988
CAD3	0.947	0.605	0.755	0.962
Microvascular disease (M)	0.938	0.629	0.310	0.986
CAD5	0.967	0.760	0.790	0.981
NORM2	0.958	0.724	0.655	0.981
Microvascular disease (F)	0.952	0.753	0.488	0.988
CAD2	0.956	0.742	0.590	0.984
CAD1	0.965	0.811	0.665	0.988
Non-coronarogenic diseases	0.957	0.722	0.650	0.981
CAD4	0.971	0.758	0.875	0.978
LVH2	0.949	0.633	0.695	0.969
NORM1	0.943	0.589	0.673	0.964

As can be seen from the result, the average accuracy of classification is 0.96, average specificity is equal to 0.98, and both average precision and sensitivity are equal to 0.7. Comparing the obtained parameters of classification with the multiclass classifier described in (9), all the parameters are higher in the case of using a two-stage classifier. Most significant increase showed sensitivity: average value is 6.4% higher. At the same time, for some of the groups, increase is even more substantial: 14.5% for the Myocardial damage group, 16.3% Microvascular disease (M), 10% for NORM2, 9.5% for Microvascular disease (F), and 12% for the Non-coronarogenic diseases group. A similar result was obtained for precision: average value is 6.8%, which is higher compared to the value obtained using a one-stage classifier. Performing two-stage classification allowed us to significantly increase the precision for two groups that had very low value: the LVH1 group by 11% and the LVH2 group by 11%.

## Conclusions

Summing up the result during the study, we can conclude that the proposed method of two-stage classification allows to improve the performance of a multiclass classifier based on correlation analysis by applying k-NN classification in determined cases with most suitable parameters. The designed two-stage classifier shows an average accuracy equal to 0.96, average specificity was 0.98, and both average precision and sensitivity are equal to 0.7. The proposed method allows to solve the problems of our previous classifier, described in (9), which are low precision for the LVH1, LVH2, LVH3, CAD3, and NORM1 groups, and low sensitivity for the NORM2, Microvascular disease (F), Microvascular disease (M), Non-coronarogenic diseases, LVH2, and CAD2 groups, increasing the corresponding values by up to 16%. Although the proposed method shows quite a good result, it can be improved even more by applying some CDDM preprocessing techniques or different features for the 2nd stage.

The proposed method of classification can help a physician to define the state of patient's cardiovascular system.

## Data Availability Statement

The raw data supporting the conclusions of this article will be made available by the authors, without undue reservation.

## Ethics Statement

The studies involving human participants were reviewed and approved by Commission on Ethics of National Military Medical Clinical Center “Main Military Clinical Hospital of Ukraine”. The patients/participants provided their written informed consent to participate in this study.

## Author Contributions

YU: method development and realization, data analysis, and experiment result analysis. AP: method review, revising mathematical background for classification approaches, experiment result analysis, and approval of obtained results from technical and mathematical side. IC: providing medical data, method review, revising medical side of data analysis, and approval of obtained results and their readiness for publication. All authors contributed to the article and approved the submitted version.

## Conflict of Interest

The authors declare that the research was conducted in the absence of any commercial or financial relationships that could be construed as a potential conflict of interest.

## Publisher's Note

All claims expressed in this article are solely those of the authors and do not necessarily represent those of their affiliated organizations, or those of the publisher, the editors and the reviewers. Any product that may be evaluated in this article, or claim that may be made by its manufacturer, is not guaranteed or endorsed by the publisher.
